# Serological survey on immunity status against polioviruses in children and adolescents living in a border region, Apulia (Southern Italy)

**DOI:** 10.1186/1471-2334-8-150

**Published:** 2008-10-30

**Authors:** Silvio Tafuri, Rosa Prato, Domenico Martinelli, Agata Calvario, Anna Bozzi, Michele Labianca, Annamaria Patti, Pietro Luigi Lopalco, Cinzia Germinario

**Affiliations:** 1 Department of Biomedical Sciences, Section of Hygiene, University of Bari, Apulia Regional Epidemiological Observatory, Bari, Italy; 2 Department of Medical Sciences, Section of Hygiene, University of Foggia, Apulia Regional Epidemiological Observatory, Foggia, Italy; 3 Microbiology and Virology Unit, WHO Sub-National Polio Reference Laboratory, Bari-Policlinico, Italy; 4 Department of Public Health Sciences, Sapienza University of Rome, Italy

## Abstract

**Background:**

In 1988 the World Health Assembly adopted the goal to eradicate poliomyelitis by routine immunization using Oral Polio Vaccine (OPV). On 21 June 2002 the WHO European Region was declared polio-free. In 2008 poliomyelitis is still endemic in 4 countries (Nigeria, India, Pakistan, and Afghanistan), where 1201 new cases were registered in 2007; 107 sporadic cases were also notified in countries where poliovirus is not endemic. The aim of this work was to verify the level of antipoliomyelitis immunity status in children and adolescents in the Apulia region (south of Italy), which may be considered a border region due to its position.

**Methods:**

704 blood specimens from a convenience sample were collected in six laboratories. The age of subjects enrolled was 0–15 years. The immunity against poliomyelitis was evaluated by neutralizing antibody titration in tissue culture microplates.

**Results:**

Seropositivity (neutralising antibodies titre ≥ 8) for polioviruses 1, 2 and 3 was detected in 100%, 99.8% and 99.4% of collected sera. Antibody titres were not lower in subjects who received either four doses of inactivated polio vaccine (IPV) or a sequential schedule consisting of two doses of IPV and two of oral polio vaccine than in subjects who received four doses of OPV.

**Conclusion:**

These results confirmed current data of vaccine coverage for poliomyelitis: during the last ten years in Apulia, the coverage in 24 months old children was more than 90%. The high level of immunization found confirms the effectiveness both of the sequential schedule IPV-OPV and of the schedule all-IPV. Apulia region has to face daily arrivals of refugees and remains subject to the risk of the importation of poliovirus from endemic areas. Surveys aimed at determining anti-polio immunity in subpopulations as well as in the general population should be carried out.

## Background

In 1988 the World Health Assembly adopted the goal to eradicate poliomyelitis by routine immunization using Oral Polio Vaccine (OPV) at birth followed by three doses at 6, 10 and 14 weeks supplemented by surveillance for acute flaccid paralysis (AFP), national immunization days (NIDs) and mopping-up immunization campaigns [1].

In developing countries, during the National Immunization Days, as recommended by the World Health Organization (WHO), two doses of OPV were given to all children under the age of five irrespective of their vaccination status [2]. The final stages of this program require a mopping-up activity, that is a door-to-door search for subjects to be immunized. This operation turns out to be particularly effective in those areas characterized by poor health infrastructure and immunization services [3-6].

The WHO European Region included in the Expanded Program of Immunization the goal of wild poliovirus global eradication [7,8]. In the European region, by the end of 1996, all poliomyelitis endemic countries had conducted NIDs (operation MEGACAR) because of a large outbreak of 154 cases of paralytic disease in Chechnya and suboptimal routine immunization coverage in other countries. In the autumn of 1996 Albania also started a mass campaign with OPV to control outbreaks [9]. In 1998 only Turkey reported 23 cases of AFP due to wild virus. Europe's last case of indigenous wild poliomyelitis occurred in eastern Turkey in 1998, when a two-year-old unvaccinated boy was paralysed by the virus. On 21 June 2002 the WHO European Region was declared polio-free.

In 2008 poliomyelitis is still endemic in 4 countries (Nigeria, India, Pakistan, and Afghanistan), where 1201 new cases were registered in 2007; 107 sporadic cases were also notified in countries where poliovirus is not endemic [10].

The WHO Polio Eradication Strategic Plan for 2004–2008 recommends the discontinuation of vaccination with OPV after global eradication of wild poliovirus [11] and the use of Inactivated Poliovirus Vaccine (IPV). IPV is considered to be safer than OPV because IPV is not associated with the rare risk of vaccine-associated paralytic poliomyelitis (VAPP) [12,13] or with the emergence of neurovirulent vaccine-derived poliovirus [14-18]. Some industrialized countries, such as Sweden, Finland and The Netherlands, have chosen to use IPV instead of OPV [19] in 2003. In the United States an all-IPV schedule has been adopted since 2000 and the use of this schedule is associated with the elimination of VAPP in USA [20].

In Italy the last two cases of poliomyelitis due to transmission of indigenous wild poliovirus occurred in 1982, when the virus was detected in subjects who had not been immunized by the age of 1 year, and the last imported case was in 1988 [21]. Vaccination against poliomyelitis with OPV was introduced in the compulsory immunization schedule in 1966: the vaccination schedule provided for three-dose administration of OPV at 3, 5 and 11 months of age and a booster dose at 3 years-old.

Since 1999, a sequential schedule consisting of two doses of inactivated polio vaccine (at 3 and 5 months) and two of oral polio vaccine (at 11 months and 3 years of age) was adopted; in July 2002 a schedule consisting of four doses of IPV was adopted [22]. In 2005, National Vaccine Plan 2005–2007 introduced a schedule consisting of four doses of IPV at 3, 5, 11 months and 4–5 years-old [23].

In 2001 in Italy a combined DTPa-HBV-IPV/Hib vaccine containing diphtheria (D), tetanus (T), acellular pertussis (Pa), hepatitis B (HBV) and types 1, 2 and 3 inactivated polioviruses (IPV) mixed with a conjugated Haemophilus influenzae type b (Hib) vaccine, administered according to a 3, 5 and 11–13 month vaccination schedule, was introduced. The availability of hexavalent vaccination should contribute to increase the vaccination coverage of the population [24] that has reached or exceeded 95% [25].

As the disease does not respect national borders or continental boundaries, regions and areas where eradication has been achieved must continue to ensure high levels of immunization coverage to prevent re-establishment of poliovirus if it is re-introduced from other countries through international travellers, migrant populations from conflict areas or population sub-groups who refuse vaccination.

Since 2002 Italy has been declared a polio-free country but the risk of poliovirus reintroduction persists because of immigration. Several seroepidemiological studies performed in the past, the last-one conducted during 1999, put in evidence unsatisfactory antibody titres in an important proportion of young-adults, specially against poliovirus 3 [26,27]. Despite the massive exodus of people from East-Europe during 1990's and the large outbreak which occurred in Albania in 1996, no imported cases have been reported in the latest period because of the good level of coverage for polio in these populations [28]. As in other countries, since 1997 the surveillance system of all AFP cases has been set up and, at the moment, it maintains a good level of sensibility [29].

The aim of this work is to estimate the immunological status against poliovirus in children and adolescents in Apulia, after almost ten years after the last study conducted [26].

## Methods

### Samples collection

During 2007, a seroepidemiological survey was conducted on blood samples collected from children and adolescents from 3 months to 15 years old. Enrolled subjects represented a convenience sample of the population applying to 6 laboratories located in five of the six Provinces of the Region (2 located in the Province of Bari, 1 in the Province of Brindisi, 1 in the Province of Foggia, 1 in the Province of Lecce; no laboratory in the Province of Taranto), for diagnostic and check up testing.

According to the present Italian Data Protection Act, informations about initials, sex and year of birth of enrolled subjects were collected; so it was impossible to assess vaccination status by card or history. Because of Data Protection Act was respected, ethical approval was not required for this study.

### Serology

Blood samples obtained from the laboratories were taken to the WHO Sub-National Polio referred Reference Laboratory in Bari and the sera stored at -20°C.

The immunity to poliovirus was measured by determining the ability of a human serum sample to neutralize the infectivity and the cytopathic effect (CPE) on cell cultures in vitro of each of the three types of poliovirus. The neutralization test in microtitre plates was performed according to the guidelines for WHO/EPI collaborative studies on poliomyelitis [30]. Serial two-fold dilution of inactivated sera (from 1:8 to 1:1024) were incubated in duplicate with 100 TCID_50_/0.025 ml challenge virus suspension of each of the three reference Sabin strains (Poliovirus type 1/Mahoney strain, Poliovirus type 2/MEF-1 strain and Poliovirus type 3/Saukett strain). After incubation for 3 hours at 36°C, 5% CO_2_, to each well with virus-serum mixture a human heteroploid Hep-2 cell suspension (1–2 × 10^4^cells/0,1 ml; MEM Earle's salts 10% FBS; 37°C, 5% CO_2_) was added. The virus titration of each strain and cell controls were added to test and incubated at 36°C for five days; the appearance of CPE was examined with an inverted microscope.

Using the Karber formula, neutralizing antibody titre (normally expressed as reciprocal) was the highest dilution of serum which protects 50% of the cultures against 100TCD_50 _of challenge virus, inhibiting the CPE. Titres ≥ 1:8 were considered positive, as recommended by WHO/EPI. Titres were computed as geometrical means (GMTs).

### Statistical analysis

Contingence tables and χ^2 ^values were used to assess differences in the immunization rates among age groups and to evaluate the association between antibody titres, age at vaccination and the vaccine schedule used for immunization. A value of p < 0.05 was considered as significant. Epi-Info 6.00 (public domain software-CDC Atlanta, Georgia; WHO Geneva) was used for statistical analysis.

## Results

704 blood samples from children and adolescents aged 3 months-15 years were collected during 2007. 52.4% (369) of the subjects were male and 47.6% (335) were female. Median age was 6.6 years; there was no statically significant difference by age between genders (p > 0,05). Table 1 shows the distribution of samples by sex and age groups. The most represented age groups were the ones in the ranges 6–10 years and 3–5 years. The subjects were from different geographical areas: 33.2% from the Province of Bari, 29.8% from the Province of BAT (Barletta, Andria, Trani), 27.7% from the Province of Lecce, 8.3% from the Province of Foggia and 1% from the Province of Brindisi.

**Table 1:** Distribution of samples by sex and age group

	**Male**		**Female**		**Total**	
	
**Age group**	***N***	***%***	***N***	***%***	***N***	***%***
< 1 year	44	11,9	29	8,7	73	10,4
1 year	23	6,2	24	7,2	47	6,7
2 years	25	6,8	18	5,4	43	6,1
3–5 years	81	21,9	77	23,0	158	22,4
6–10 years	116	31,4	117	34,9	233	33,1
11–12 years	39	10,6	31	9,3	70	9,9
13–15 years	41	11,1	39	11,6	80	11,4
Total	369		335		704	

**Figure 1:**
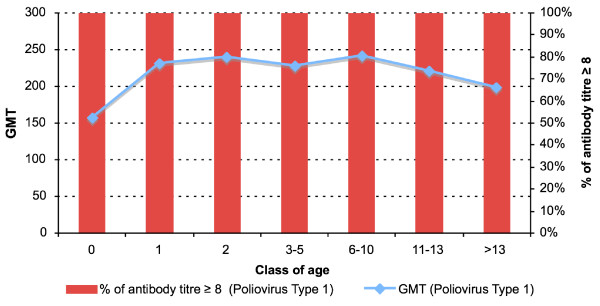
**Antibody titres against Poliovirus Type 1.** Distribution by age.

**Figure 2:**
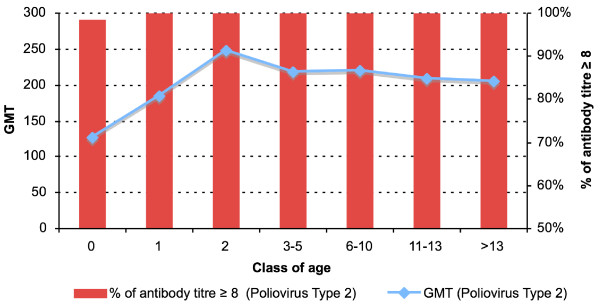
**Antibody titres against Poliovirus Type 2.** Distribution by age.

**Figure 3:**
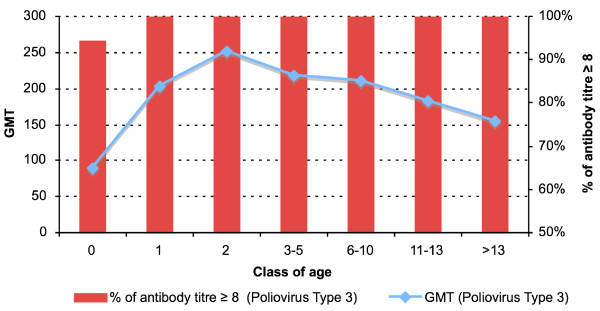
**Antibody titres against Poliovirus Type 3**. Distribution by age.

An antibody titre ≥ 1:8 was found in 704 subjects (100%) for poliovirus type 1 (Figure 1), in 703 subjects (99.8%) for poliovirus type 2 (Figure 2) and in 700 subjects (99.4%) for poliovirus type 3 (Figure 3). The protection rate for all poliovirus types was 100% in all age group, whereas the protection rate for poliovirus type 2 was 98.6% and for poliovirus type 3 was 95% in the subjects < 1 year-old.

By dividing the antibody titres into two ranges (low: 8–32; high: 64 – > 256) the number of subjects with titres 64 – > 256 was higher than that of subjects with titres 8–32 in all age groups and for all three types of polioviruses (for poliovirus type 1: χ^2 ^= 42.6; p < 0.001-for poliovirus type 2: χ^2 ^= 27.6; p < 0.001-for poliovirus type 3: χ^2 ^= 59.8; p < 0.001).

Antibody titres 8–32 for poliovirus type 1 were associated with low titres for poliovirus type 2 (OR = 37.3; 95% CI: 10.5–133.1; χ^2 ^= 74.95; p < 0.001) and for poliovirus type 3 (OR = 20.6; 95% CI: 6.3–67.5; χ^2 ^= 47.6; p < 0.001). Antibody titres 8–32 for poliovirus type 2 were also associated with low titres for poliovirus type 3 (OR = 22.6; 95% CI: 8.3–61.3; χ^2 ^= 71.9; p < 0.001).

The age < 1 year was associated with antibody titres 8–32 for all three types of polioviruses (for poliovirus 1: OR = 19.3; 95% CI: 5.6–65.8; χ^2 ^= 47.6; p < 0.001-for poliovirus 2: OR = 7.6; 95% CI: 2.9–20.0; χ^2 ^= 23.0; p < 0.001-for poliovirus 3: OR = 8.8; 95% CI: 4.4–17.7; χ^2 ^= 51.0; p < 0.001).

Antibody titres higher than 8–32 for all three types of polioviruses were detected in subjects aged > 1 year who most likely received a vaccination schedule providing three-dose administration, as all-IPV schedule (aged 1–2 years), as sequential schedule (aged 3–8 years) or an all-OPV schedule (aged 9–15 years).

## Discussion

The results of this survey showed a good level of immunity against polioviruses 1, 2, 3 in Apulian children and adolescents and this agrees with other serological surveys performed in some Italian regions in the last decade [31-34].

The lowest antibodies titres were detected in infants (age < 1 year) who could not complete the primary vaccination schedule. Antibodies titres against poliovirus 3 were > 95%, showing an improvement on the last studies in which neutralizing antibodies against poliovirus type 1, 2 and 3 were detected in 97.6%, 95.8% and 70% of samples respectively, and 3/1000 (0.3%) subjects were simultaneously seronegative to the three types [26,27].

These results confirmed current data of vaccine coverage for poliomyelitis: during the last ten years in Apulia, the coverage in 24 months old children was more than 90% [25].

Even if in this study we did not assess immunization status diretly and than we did not know if enrolled subjects received one, two or three doses of vaccine or any, the high level of immunization found could confirm the effectiveness both of the sequential schedule IPV-OPV and of the schedule all-IPV [16]. Our study shows strong evidence that the changeover from an all-OPV schedule to one containing all-IPV was not associated with a negative impact on immunization coverage. In fact, receiving all-IPV or a sequential vaccination schedule was not associated with lower antibodies titres.

As vaccine induced immunity declines with age, priority in our country is to sustain high routine immunization coverage and to emphasize a timely completion of primary immunization in accordance with the vaccine schedule.

A limitation of this study concerns sample enrollement: subjects who go to a laboratory are likely receiving medical care by definition, and might be more likely to be vaccinated than those who did not go to a laboratory.

We can not exclude the existence of low-immunity pockets in the population that were not detected in our study. Surveys aimed at determining anti-polio immunity in subpopulations as well as in the general population should be carried out; the presence of population groups with suboptimal coverage can alter the herd immunity protection.

## Conclusion

The Apulia region, which may be considered a border region due to its position, has to face daily arrivals of refugees and remains subject to the risk of the importation of poliovirus from endemic areas. Moreover, prisoners could be at risk for a not-enough coverage.

Since Italy has obtained the certification of a "polio-free" country, it is very important to prevent any possible risk of introducing the wild polioviruses. This goal will be reached by improving three strategies: the Acute Flaccid Paralysis active surveillance system, research on environmental wild polioviruses and by measures aiming at evaluating and improving vaccine coverage against poliomyelitis in children and immigrants.

As considered by Fine PE et al. in 2004, now that the global eradication of wild poliovirus is almost within sight, planning for the post-certification era is becoming a priority issue. It is agreed that a stockpile of appropriate polio vaccines will need to be established, and a surveillance and response capacity will need to be maintained, in order to protect the world against any possible future outbreaks attributable either to the persistence of wild poliovirus or vaccine-derived polioviruses or to the unintentional or intentional release of poliovirus from a laboratory or vaccine store. Although it has been suggested that the stockpile should consist of monovalent oral poliovirus vaccine (mOPV), many questions remain concerning its nature, financing, management and use, in particular because of uncertainties over future national vaccination policies and over the availability of different vaccines after the certification of wild poliovirus eradication. There are further uncertainties concerning the possible role and efficacy of IPV used in outbreak control in low-hygiene settings, the potential for rapid geographical spread of polioviruses should an outbreak occur after certification, and the risks inherent in introducing additional OPV viruses into populations in which the vaccine coverage and prevalence of immunity have declined, and which may thus favour the spread of VDPVs. Given these important gaps in knowledge, no country should discontinue polio vaccination until a coordinated policy for the post-certification era has been developed and the recommended measures have been put in place [35].

## List of abbreviations

**AFP**: acute flaccid paralysis; **DTPa-HBV-IPV/Hib**: vaccine containing diphtheria (D), tetanus (T), acellular pertussis (Pa), hepatitis B (HBV) and types 1, 2 and 3 inactivated polioviruses (IPV) mixed with a conjugated Haemophilus influenzae type b (Hib); **IPV**: inactivated polio vaccine; **mOPV**: monovalent oral poliovirus vaccine; **NIDs**: national immunization days; **OPV**: Oral Polio Vaccine; **VAPP**: vaccine-associated paralytic poliomyelitis; **WHO**: World Health Organization.

## Competing interest

The authors declare that they have no competing interests.

## Authors' contributions

ST and RP conceived of the study, partecipated in its design, performed analysis and interpretation of data and drafted the manuscript. DM contributed to analysis and interpretation of data. AC and AB carried out the virological analyses and partecipated in virological protocol drafting. AP contributed to conception of the study. PL e CG conceived of the study and contributed to interpretation of data.

## Pre-publication history

The pre-publication history for this paper can be accessed here:


